# Confidence interval estimation for vaccine efficacy against COVID-19

**DOI:** 10.3389/fpubh.2022.848120

**Published:** 2022-08-12

**Authors:** Qinyu Wei, Peng Wang, Ping Yin

**Affiliations:** Department of Epidemiology and Biostatistics, School of Public Health, Tongji Medical College, Huazhong University of Science and Technology, Wuhan, China

**Keywords:** vaccine efficacy, fixed number of events design, under-sensitivity, COVID-19, coverage probability

## Abstract

This article focuses on the construction of a confidence interval for vaccine efficacy against contagious coronavirus disease-2019 (COVID-19) in a fixed number of events design. Five different approaches are presented, and their performance is investigated in terms of the two-sided coverage probability, non-coverage probability at the lower tail, and expected confidence interval width. Furthermore, the effect of under-sensitivity of diagnosis tests on vaccine efficacy estimation was evaluated. Except for the exact conditional method, the non-coverage probability of the remaining methods may exceed the nominal significance level, e.g., 5%, even for a large number of total confirmed COVID-19 cases. The narrower confidence interval width from the Bayesian, approximate Poisson, and mid-P methods are on the cost of increased instability of coverage probability. When the sensitivity of diagnosis test in the vaccine group is lower than that in the placebo group, the reported vaccine efficacy tends to be overly optimistic. The exact conditional method is preferable to other methods in COVID-19 vaccine efficacy trials when the total number of cases reaches 60; otherwise, mid-p method can be used to obtain a narrower interval width.

## Introduction

The contagious coronavirus disease-2019 (COVID-19) pandemic continues to present a challenge to global health. There is no effective treatment or cure for the disease, and vaccination remains the most effective method to block the rapid spread of the virus for the near future. Many COVID-19 vaccine developers have published their vaccine efficacy (VE) results against symptomatic COVID-19 from ongoing phase 3 trials (1–6). According to the Food and Drug Administration (FDA) issued guidance, the statistical success criterion for a placebo-controlled efficacy trial should be that the point estimate of VE is at least 50%, and the lower bound of the Confidence Interval (CI) around the VE point estimate is > 30% (7). Different CI estimation methods are available for VE, which may lead to different lower limits, influencing the final conclusion.

In vaccine field efficacy trials where the prevalence of disease is low, VE is always estimated by one minus the incidence rate ratio (vaccine group vs. placebo group), with the incidence rate calculated as the number of infected cases divided by the total person time at risk in each group (8). Such studies are generally designed to accrue a fixed number of infected cases rather than running a fixed surveillance period for each subject. Conditional on the total number of confirmed COVID-19 cases, the CI for VE can be constructed from the Binominal or Poisson distribution, adjusting for the surveillance time. The discrete nature of the Binomial and Poisson distributions make it impossible in many situations to precisely attain the desired significance level. The coverage probability of interval estimation for a single Binomial proportion has been widely investigated (9–13), but few studies have investigated the performance of these methods on constructing VE intervals. Ewell (1996) suggested that the exact conditional method is too conservative, and large sample approximation method or Bayesian method can be used instead to obtain a narrower interval width (14). In their study, the Wald method was used for approximation. However, many studies have shown that the Wald method is unstable for the interval estimation of a single proportion (11–13).

VE rate plays a crucial role in public health planning. This study presented five VE interval estimation methods and evaluated their performance based on two-sided coverage probability, non-coverage probability at the lower tail, and expected interval width. We also investigated the effect of under-sensitivity from imperfect diagnosis test on the estimation of VE and corresponding CI. Finally, we illustrate these methods using published data from phase III COVID-19 vaccine trials.

## Materials and methods

In trials where the incidence of the disease is measured by incidence rates, VE can be estimated as $\hat{VE}=1-IRR$ where IRR being the estimated incidence rate ratio, which is calculated as the incidence rate in the investigational vaccine group vs. that in the placebo group. Let *c*_1_, *T*_1_, *c*_0_, and *T*_0_ denote the number of infected cases and total person-time at risk in the investigational vaccine and placebo groups, respectively. The incidence rate in each group is estimated by dividing the number of cases diagnosed by the total person-time at risk of that group, where the total person-time at risk is the sum of the individual person-time at risk, which is the length of surveillance period for non-infected subjects, time to infection for infected subjects, and time to discontinuation for non-infected dropouts. IRR can be calculated as $IRR=\;\frac{c_{1}/T_{1}}{c_{0}/T_{0}}$.

### Intervals based on conditional binomial distribution

Assuming that the number of cases during the surveillance period for each group follows a Poisson distribution, with parameter λ_1_ for the investigational vaccine group and λ_0_ for the placebo group, then *c*_1_ is binomially distributed *B*(*c*, π) conditional on the total number of cases *c* where *c* = *c*_0_+*c*_1_, and with $\pi =\frac{T_{1}\lambda_{1}}{T_{1}\lambda_{1}+T_{0}\lambda_{0}}$. The relationship between π and VE is expressed as


(1) $$\begin{array}{l} VE=1-IRR=1-\frac{c_{1}/T_{1}}{c_{0}/T_{0}}=1-\frac{\lambda_{1}}{\lambda_{0}}=1-\frac{\pi }{r(1-\pi )} \end{array}$$

Where, the constant *r* = *T*_1_/*T*_0_ is the ratio of the total person-time at risk in the vaccine and placebo groups.

The interval estimation for VE is then converted to the problem of constructing CI for a single Binomial proportion of π. If (*L*_π_, *U*_π_) is the 100(1−α)% CI for π, then the 100(1−α)% CI for VE can be derived as:


(2) $$\begin{array}{l} \left(1-\frac{U_{\pi }}{r\left(1-U_{\pi }\right)},\;1-\frac{L_{\pi }}{r\left(1-L_{\pi }\right)}\right). \end{array}$$

#### Exact conditional interval

The Clopper-Pearson interval for π is constructed by inverting the equal-tailed test based on the binomial distribution (15). The upper and lower exact confidence limits (*L*_π_, *U*_π_) satisfy the following equations.


$$\begin{array}{l} \overset{n}{\underset{x=c_{1}}{\sum }}\left(\begin{array}{c} c\\ x \end{array}\right){L_{\pi }}^{x}{\left(1-L_{\pi }\right)}^{c-x}=\;\alpha /2\\ \overset{c_{1}}{\underset{x=0}{\sum }}\left(\begin{array}{c} c\\ x \end{array}\right){U_{\pi }}^{x}{\left(1-U_{\pi }\right)}^{c-x}=\;\alpha /2 \end{array}$$

Using the relationship between the binomial summations and beta integrals, (*L*_π_, *U*_π_) can be expressed as quantiles of the following Beta distributions.


(3) $$\begin{array}{l} \left[Beta\left(\frac{\alpha }{2},c_{1},c-c_{1}+1\right),\;Beta\left(1-\frac{\alpha }{2},c_{1}+1,c-c_{1}\right)\right] \end{array}$$

The interval for π can then be converted into the CI for VE by using Equation (2).

#### Mid-p interval

The mid-p approach replaces the probability of the observed frequency by half of that probability in the Clopper-Pearson sum, leading to an “approximate” interval. This family of intervals aims for mean coverage to be close to nominal level without compromising minimum coverage too much. The exact mid-p confidence limits *L*_π_ and *U*_π_ are solutions to the following equations.


$$\begin{array}{l} Pr_{U_{\pi }}\left(x<c_{1}\right)+0.5\times Pr_{U_{\pi }}\left(x=c_{1}\right)=\;\alpha /2\\ Pr_{L_{\pi }}\left(x>c_{1}\right)+0.5\times Pr_{L_{\pi }}\left(x=c_{1}\right)=\;\alpha /2 \end{array}$$

The interval for π can then be converted into the CI for VE by using Equation (2).

### Bayesian interval with beta conjugate prior

Assume that π follows a prior beta distribution *beta*(*a, b*) with parameters *a* and *b*. The mean of the prior distribution is *u* = *a*/(*a*+*b*) and the variance is *u*(1−*u*)/(*a*+*b*+1). The posterior distribution of π given *c*_1_ infected cases in the vaccine group conditional on the total number of cases *c* is again a member of the beta family, denoted *beta*(*a* + *c*_1_, *b* + *c* − *c*_1_). The resulting limits of the Bayesian interval are the α/2^th^ and (1−α/2)^th^ quantiles of the posterior *beta* distribution, respectively. The 100(1- α)% equal tailed Bayesian interval is given by


(4) $$\begin{array}{l} \left[Beta\left(\frac{\alpha }{2};a+c_{1},b+c-c_{1}\right),Beta\left(1-\frac{\alpha }{2};a+c_{1},b+c-c_{1}\right)\right]. \end{array}$$

Notably, when *a* = *b* = 0.5, the above interval corresponds to the 100(1- α)% equal-tailed Jeffreys interval.

According to Pfizer's protocol, a minimally informative beta prior, beta (0.700102, 1), which is centered at θ = 0.4118 (VE=30%), was applied to construct the posterior probability for π (16). The 100(1- α)% equal tailed interval for π is given by


(5) $$\begin{array}{l} {}[Beta(\frac{\alpha }{2};c_{1}+0.700102,c-c_{1}+1),\\ Beta(1-\frac{\alpha }{2};c_{1}+0.700102,c-c_{1}+1)]. \end{array}$$

The interval for π can then be converted into the CI for VE by using Equation (2).

### Approximate poisson interval

This method is based on normal approximation for a logarithmic transformation of the incidence rate ratio, the variance of log(*IRR*) can be written as the sum of variances of the log incidence rates in vaccine group and placebo group.


$$\begin{array}{l} var\left(\log IRR\right)=var\left[\log\left(\frac{c_{1}/T_{1}}{c_{0}/T_{0}}\right)\right]=var\left[\log\left(c_{1}/T_{1}\right)\right]\\ \;+\;var\left[\log\left(c_{0}/T_{0}\right)\right] \end{array}$$

By a Taylor's series approximation, the variance of log(*c*_*x*_/*T*_*x*_) is $var\left(c_{x}\right)/{c_{x}}^{2}$, and since *c*_*x*_ follows a Poisson distribution, an estimate of *var*(*c*_*x*_) is given by *c*_*x*_. This yields the following large sample two-sided 100(1−α)% CI for the incidence rate ratio (14):


$$\begin{array}{l} \exp\left\{\log\left(IRR\right)\pm z_{1-\frac{a}{2}}\sqrt{\frac{1}{c_{1}}+\frac{1}{c_{0}}}\right\} \end{array}$$

The above CI can also be obtained from the Poisson regression using the maximum likelihood estimate (MLE) of the parameters with treatment as a fixed effect and time at risk for each subject as offset in the model.

The 100(1−α)% CI for VE can then be expressed as:


(6) $$\begin{array}{l} \left[1-IRRexp(z_{1-\frac{a}{2}}\sqrt{\frac{1}{c_{1}}+\frac{1}{c_{0}}}),\;1-RRexp(-z_{1-\frac{a}{2}}\sqrt{\frac{1}{c_{1}}+\frac{1}{c_{0}}})\right] \end{array}$$

When *c*_1_ = 0 or *c*_0_ = 0, the approximate Poisson interval doesn't exist.

## Criteria for evaluation

To evaluate the performance of the 95% CI constructed using the preceding methods, simulations were conducted to compare the two-sided coverage probability (CP), non-coverage at lower tail (NCL), and expected interval width. In addition, the effect of under-sensitivity from imperfect diagnostic test for COVID-19 were investigated.

A variety of scenarios were simulated with null VE from 0.5 to 1 in the increments of 0.001, and the total number of cases c was set to be 10, 20, 60, and 500. The 2-sided significance level α was fixed at 0.05. For easy of comparison, we fixed the ratio of surveillance time in vaccine group to placebo group to be 1. Similar conclusion can be made when *r*≠1. Approximate Poisson method cannot provide interval for boundary outcomes for which the exact conditional interval is applied at *c*_1_ = 0 and *c*_1_ = *c*. To investigate the effect of under-sensitivity of the diagnosis test on the observed VE interval, we fixed the sensitivity in the placebo group at *s*_1_ = 100%, and set the ratio of sensitivity *s*_1_/*s*_0_ ranging from 0.9 to 1 in increments of 0.02.

All the results were generated using R version 4.1.1. Below are definitions for these evaluation criteria.

### Two-sided coverage probability

At a fixed vaccine efficacy *ve* with given *c*, *r*, and *a*, the coverage probability is defined as the proportion of the two-sided (1−α) CI containing the true *ve*. An ideal interval estimation method has the coverage probability equal to (1−*a*) under all parameter settings, e.g., a 95% CI contains the true *ve* with 95% probability. However, this is not always the case for data that follow discrete distributions.

Given the total number of cases *c*, the possible combinations of the number of cases in the vaccine group *c*_1_ = 0, 1, 2, …, *n* and the number of cases in the placebo group *c*_0_ = *c*−*c*_1_ are exhaustive. The binomial probability for each combination of (*c*_1_, *c*_0_) is given by $\left(\begin{array}{c} c\\ c_{1} \end{array}\right)\pi^{c_{1}}{\left(1-\pi \right)}^{c-c_{1}}$ where $\pi =1-\frac{1}{1+\left(1-VE\right)r}$ according to Equation (1). The coverage probability can then be estimated by summing the probabilities of all combinations for which the resulting interval contains the true *ve*. For each configuration of (*ve, c, r, a*), the coverage probability can be calculated using the following formula.


(7) $$\begin{array}{r} CP=\sum_{k=0}^{c}I\left(k,ve\right)\left(\begin{array}{c} c\\ k \end{array}\right)\\ {\left(1-\frac{1}{1+\left(1-ve\right)r}\right)}^{k}{\left(\frac{1}{1+\left(1-ve\right)r}\right)}^{c-k} \end{array}$$

where *I*(*k, ve*) is an indicator variable that equals to 1 if the CI includes *ve* when *c*_1_ = *k*, otherwise *I*(*k, ve*) = 0.

### Non-coverage probability at the lower tail

VE is always demonstrated by comparing the lower limit of the CI with a given margin. When the non-coverage probability at the lower tail is greater than the nominal level *a*/2, it may lead to inflation of the type I error rate. Owing to the asymmetry of the Poisson and Binomial distributions, the non-coverage probability at the two tails is always unequal. For a given method under each configuration of (*ve, c, r, a*), the non-coverage probability at the lower tail can be calculated as the sum of the binomial probabilities of all combinations for which the lower limit of the resulting CI is greater than the true vaccine efficacy *ve*.


(8) $$\begin{array}{l} NCL=\sum_{k=0}^{c}J\left(k,ve\right)\left(\begin{array}{c} c\\ k \end{array}\right){\left(1-\frac{1}{1+\left(1-ve\right)r}\right)}^{k}\\ \;{\left(\frac{1}{1+\left(1-ve\right)r}\right)}^{c-k} \end{array}$$

Where, indicator *J*(*k, ve*) = 1 if the lower limit of the CI is greater than the true *ve* when *c*_1_ = *k*, otherwise *J*(*k, ve*) = 0.

### Expected interval width

The expected interval width can be calculated using a formula similar to that of the coverage probability and non-coverage probability. For each configuration of (*ve, c, r, a*), it can be calculated as follows.


(9) $$\begin{array}{l} Len=\sum_{k=0}^{c}Len\left(k,ve\right)\left(\begin{array}{c} c\\ k \end{array}\right){\left(1-\frac{1}{1+\left(1-ve\right)r}\right)}^{k}\\ \;{\left(\frac{1}{1+\left(1-ve\right)r}\right)}^{c-k} \end{array}$$

where *Len*(*k, ve*) is the interval width of the resulting CI when *c*_1_ = *k*. Because *ve* ranges from −∞ to 100%, the interval width was set to 2 when the lower limit is lower than −100%; otherwise, it was calculated as (upper limit—lower limit).

### Under-sensitivity of the diagnosis test

Reported VE always implicitly assumes that the diagnostic test has a sensitivity and specificity of 100%. However, this assumption is invalid according to the reported sensitivity and specificity of COVID-19 diagnosis testing (17, 18). The estimated VE can be biased by an unknown amount when the sensitivity or specificity of the diagnostic test is <100% (19, 20). Diagnostic tests are rarely totally accurate. We assume that the diagnosis of COVID-19 with a specificity of 100%. However, the sensitivity for identifying a COVID-19 infection varies based on the type and quality of the specimen obtained and duration of illness at the time of testing (21). Ridgway et al. (18) reported that sensitivity of a single NAAT test ranged from 82 to 97% among symptomatic patients utilizing 34348 SARS CoV-2 NAAT results from two health systems (18).

The sensitivity of a diagnostic test is the conditional probability that the test will be positive (Test+) if the disease is present (Disease+).


$$\begin{array}{l} sensitivity=Pr\left(Test+|Disease+\right), \end{array}$$

Specificity is the conditional probability that the test will be negative (Test–) if the disease is not present (Disease–).


$$\begin{array}{l} specificity=Pr\left(Test-|Disease-\right). \end{array}$$

When the sensitivity and specificity of the test are known, the adjusted number of cases, also called the actual number of cases *C*_*actual*_, is calculated according to the following formula.


$$\begin{array}{l} C_{actual}=\frac{C_{observed}+Sp-1}{Se+Sp-1} \end{array}$$

Where, *C*_*observed*_, *Se*, and *Sp* denote the observed number of cases, sensitivity, and specificity, respectively.

When the specificity is 100%, the above formula can be written as *C*_*actual*_ = *C*_*observed*_/*Se*.

Let *s*_1_ and *s*_0_ denote the sensitivities in the Vaccine and placebo groups, respectively. The number of the observed cases in the vaccine and placebo groups can then be derived as *c*_1*o*_ = *c*_1_*s*_1_ and *c*_0*o*_ = *c*_0_*s*_0_, respectively. The impact of under-sensitivity of diagnosis test on the non-coverage probability at the lower tail and expected lower limit are investigated. The expected lower limit is calculated in a similar way with expected interval width.

### Example COVID-19 vaccine efficacy trials

The reported vaccine efficacy data from the following three COVID-19 studies will be used to illustrate our results. The examples are sorted by the vaccine name in an alphabetical order.

Example 1 (BNT162b2 vaccine): This is an ongoing multinational, placebo-controlled, observer-blinded, pivotal efficacy among persons 16 years of age or older. Participants were randomized in a 1:1 ratio to receive two doses, 21 days apart, of either placebo (*N* = 17511) or the BNT162b2 vaccine (*N* = 17411). The primary endpoint is VE against confirmed Covid-19 with onset at least 7 days after the second dose. There were 170 cases of symptomatic COVID-19 included in the primary efficacy analysis (3).

Example 2 (ChAdOx1 vaccine): This is an ongoing single-blind phase 3 trial in Brazil among adults 18 years and older. Participants were randomized to control group (*N* = 2025) or ChAdOx1 group (*N* = 2063) in a 1:1 ratio. All participants were offered 2 doses with administration 4 weeks apart. The primary endpoint is VE against symptomatic COVID-19 more than 14 days after the second dose of vaccine. There were 131 cases of symptomatic COVID-19 included in the primary efficacy analysis (4).

Example 3 (HBO2 vaccine): This is an ongoing randomized, double-blind, phase 3 trial in the United Arab Emirates and Bahrain among adults 18 years and older. Participants were randomized to receive 1 of 2 inactivated vaccines developed from WIV04 (*N* = 13 459) and HB02 (*N* = 13 465) strains or Placebo (*N* = 13 458); they received 2 intramuscular injections 21 days apart. The primary endpoint is VE against laboratory-confirmed symptomatic COVID-19 that occurred at least 14 days after a second vaccine dose. There were 142 symptomatic COVID-19 cases (95 cases in Placebo group; 26 cases in WIV04 group; 21 cases in HB02 group) and we randomly selected HB02 group for the analysis (6).

## Results

### Oscillation behavior of coverage probability for VE intervals

**Figure 1:**
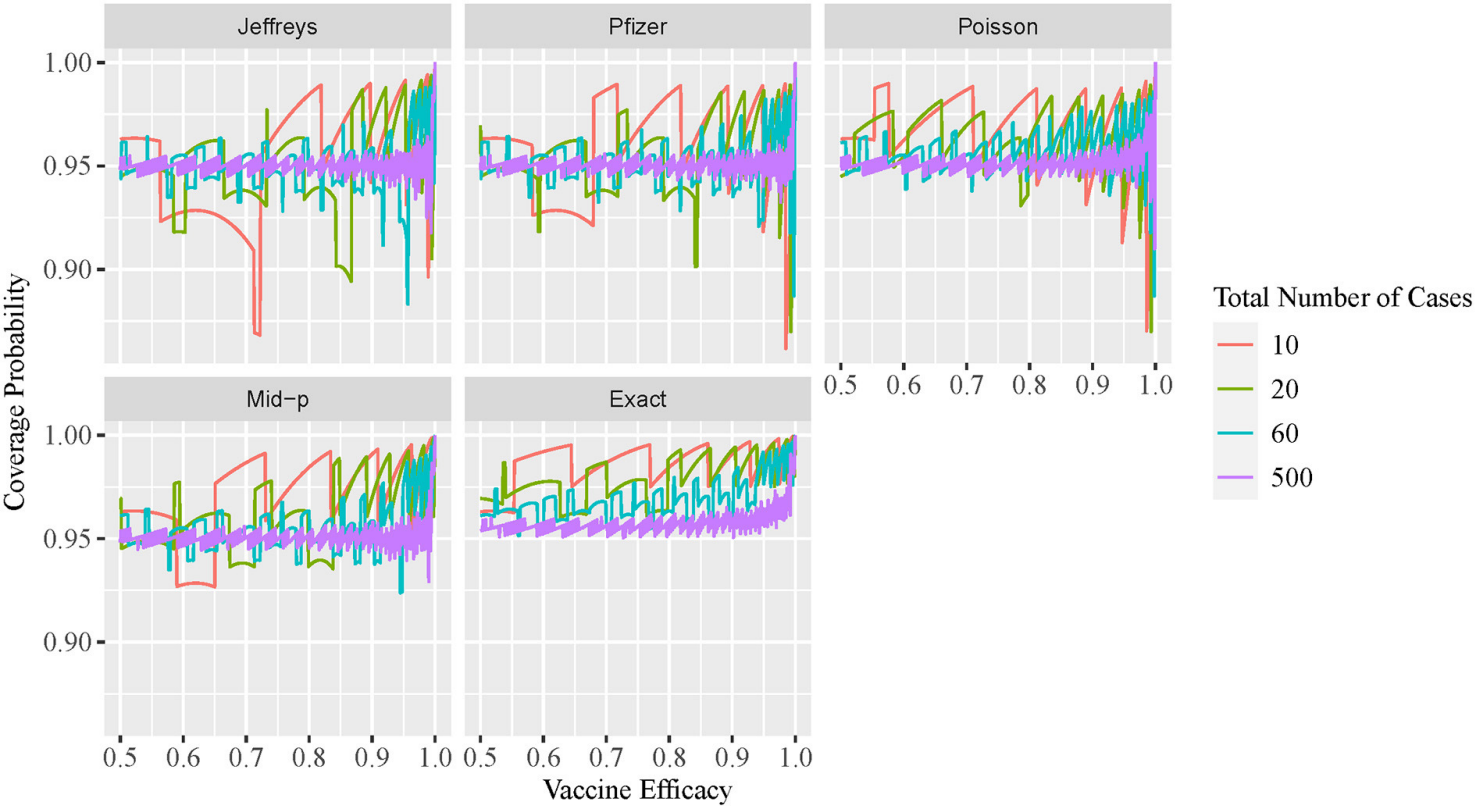
Line plot of coverage probability for different VE intervals.

**Figure 2:**
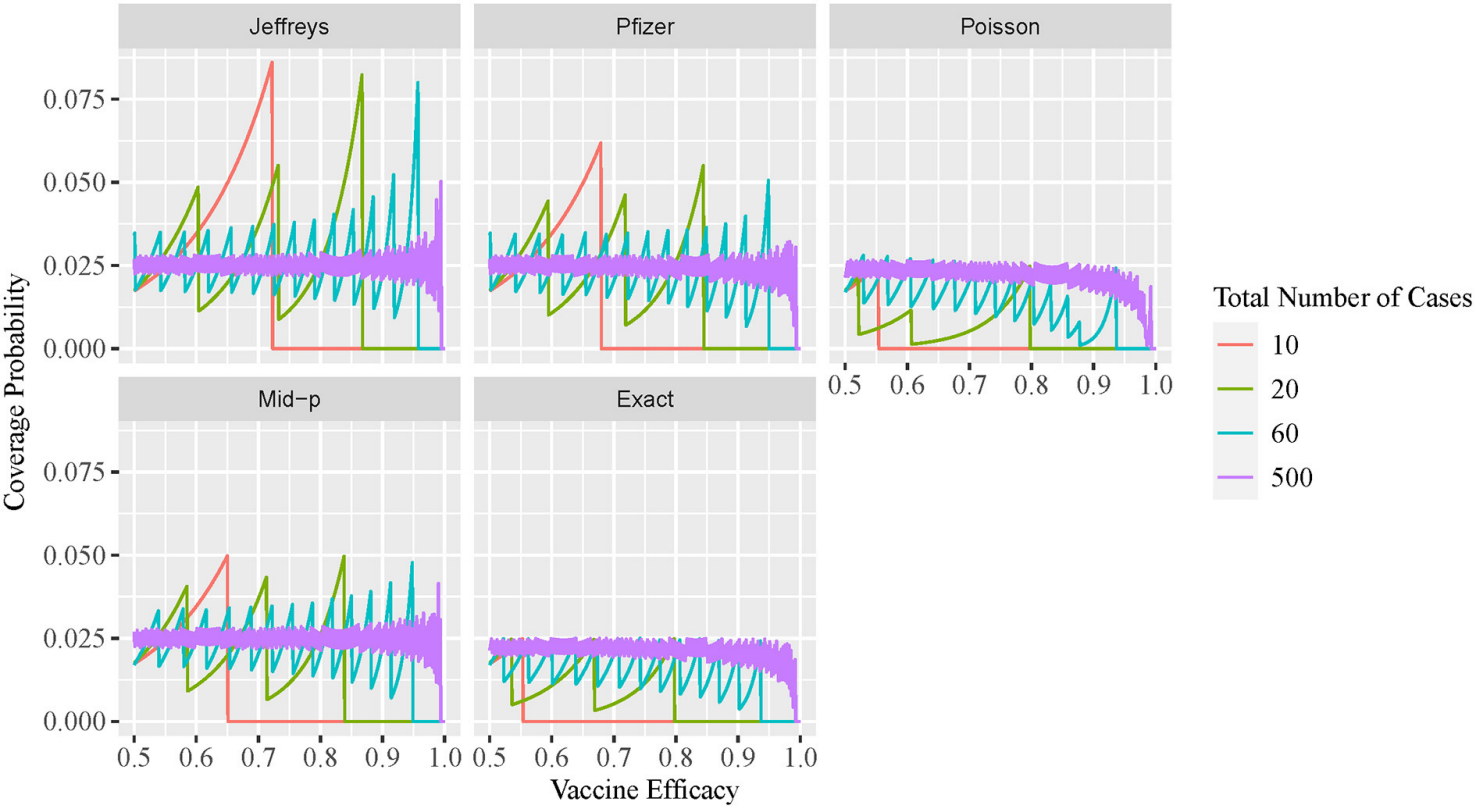
Line plot of non-coverage probability at lower tail for different VE intervals.

The two-sided coverage probability over the *ve* overlapped by *c* are presented in Figure 1 using line plots with the horizontal and vertical axes indicating the *ve* and coverage probability, respectively. Similarly, the non-coverage probability at the lower tail is plotted in Figure 2. In addition, a descriptive summary of the coverage probability and non-coverage probability at the lower tail is provided in Table 1.

**Table 1:** Summary of coverage probability, non-coverage at lower tail, and expected interval width.

**Method**	**Total Cases**	**Coverage probability (%)**		**Non-coverage at lower tail (%)**		**Median expected interval width**
		**Mean**	**Minimum**	**Mean**	**Maximum**	
Jeffreys	10	95.4	86.8	1.9	8.6	1.01
	20	95.1	89.4	2.2	8.2	0.64
	60	95.0	88.3	2.4	8	0.33
	100	95.0	88.1	2.5	7.7	0.25
	300	95.0	91.3	2.5	6.8	0.14
	500	95.0	91.8	2.5	5	0.11
Pfizer	10	96.0	86.2	1.2	6.2	1.00
	20	95.5	87.0	1.7	5.5	0.63
	60	95.2	88.7	2.1	5.1	0.33
	100	95.1	90.5	2.2	4.7	0.25
	300	95.1	91.5	2.4	3.9	0.14
	500	95.0	93.2	2.4	3.2	0.11
Approximate Poisson	10	96.8	87.0	0.2	2.5	1.17
	20	96.4	87.0	0.5	2.5	0.70
	60	95.7	88.7	1.3	2.8	0.34
	100	95.5	90.5	1.6	2.8	0.26
	300	95.2	93.6	2	2.7	0.14
	500	95.1	91.0	2.1	2.7	0.11
Mid-p	10	97.1	92.7	0.9	5	1.09
	20	96.2	93.5	1.6	5	0.67
	60	95.5	92.4	2.1	4.8	0.34
	100	95.3	92.1	2.3	4.7	0.25
	300	95.1	93.0	2.4	3.9	0.14
	500	95.1	92.9	2.4	4.1	0.11
Exact	10	98.6	96.3	0.2	2.5	1.24
	20	97.9	96.0	0.7	2.5	0.75
	60	96.9	95.1	1.4	2.5	0.36
	100	96.5	95.0	1.6	2.5	0.27
	300	96.0	95.1	2	2.5	0.15
	500	95.8	95.0	2.1	2.5	0.11

As shown in Figure 1, the coverage probability is not fixed at the nominal level 1−α = 95% for all methods. As *c* increases, the coverage probability approaches the nominal level 95%, and oscillation decreases but still exists even for large values of *c*. Under all parameter settings, the exact conditional method has a two-sided coverage probability on or above the nominal level 95%. It is clear from the plot that the oscillatory is more significant for Bayesian intervals using either the non-informative Jeffreys prior or minimal informative Pfizer prior, when compared to the other three methods. As indicated in Table 1, the minimum coverage is 86.8% for the Bayesian method with the Jeffreys prior and 86.2% for the Pfizer prior. The Pfizer method performs slightly better than the Jeffreys prior and its low-coverage mainly comes from the region where the true *ve* is close to 100% when *c* reaches 60. The approximate Poisson method has minimum coverage below 90% when the true *ve* is close to 100% and above 92.5% when the true *ve* is not close to 100%. The mid-p interval has minimum coverage probability above 92.5% under all scenarios.

As shown in Figure 2, the non-coverage probability of the exact conditional method is no greater than the nominal level α/2 = 2.5% under all scenarios. As shown in Figure 2, the Bayesian intervals using either non-informative Jeffreys prior or minimal informative Pfizer prior has a higher non-coverage probability at the lower tail, when compared to the other three methods. The non-coverage probability can be three times higher than the nominal level 2.5% for the Jeffreys prior, and two times higher than the nominal level 2.5% for the Pfizer prior. For the mid-p method, the non-coverage probability at the lower tail is always below 5%. The non-coverage probability of the approximate Poisson interval at the lower tail is desirable, with a maximum value of 2.8% as shown in Table 1. However, the coverage probability of Poisson method is low when the *ve* is close to 1, as shown in Figure 1, which indicates that the non-coverage of Poisson method mainly comes from the upper tail.

### Comparison of expected interval width

**Figure 3:**
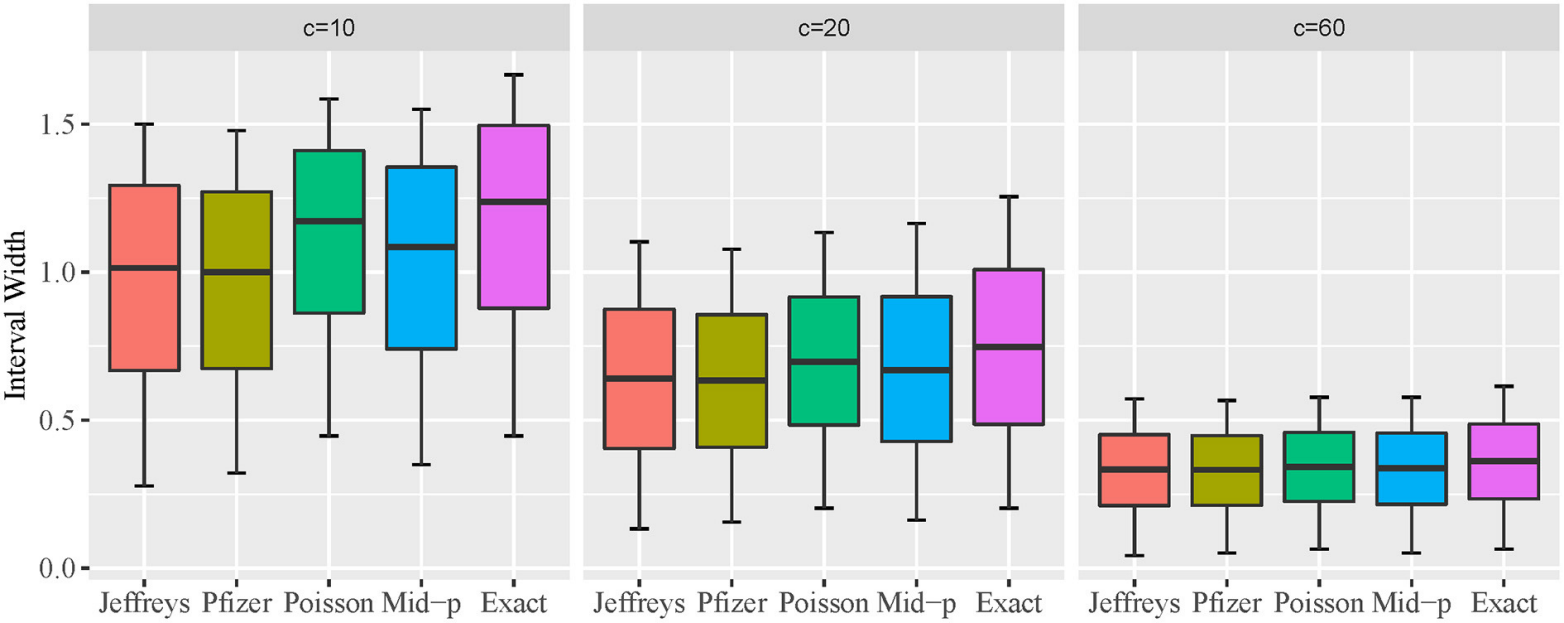
Box plot of expected two-sided interval width for different VE intervals.

The expected interval width of two-sided 95% CI constructed by the five methods for *c* = 10, 20, *and* 60, and VE from 0.5 to 1 is presented in Figure 3 using box plot. A descriptive summary of the expected interval width is provided in Table 1. The exact conditional and approximate Poisson methods provide wider interval widths under all scenarios, and the approximate Poisson method has slightly narrower interval compared to the exact conditional method. Bayesian methods are consistently narrower than the other methods. The pattern of the interval widths for all five methods become increasingly more similar as *c* increases. When *c* reaches 60, the interval width of all five methods is very close.

### Effect of diagnosis test sensitivity on VE estimation

**Figure 4:**
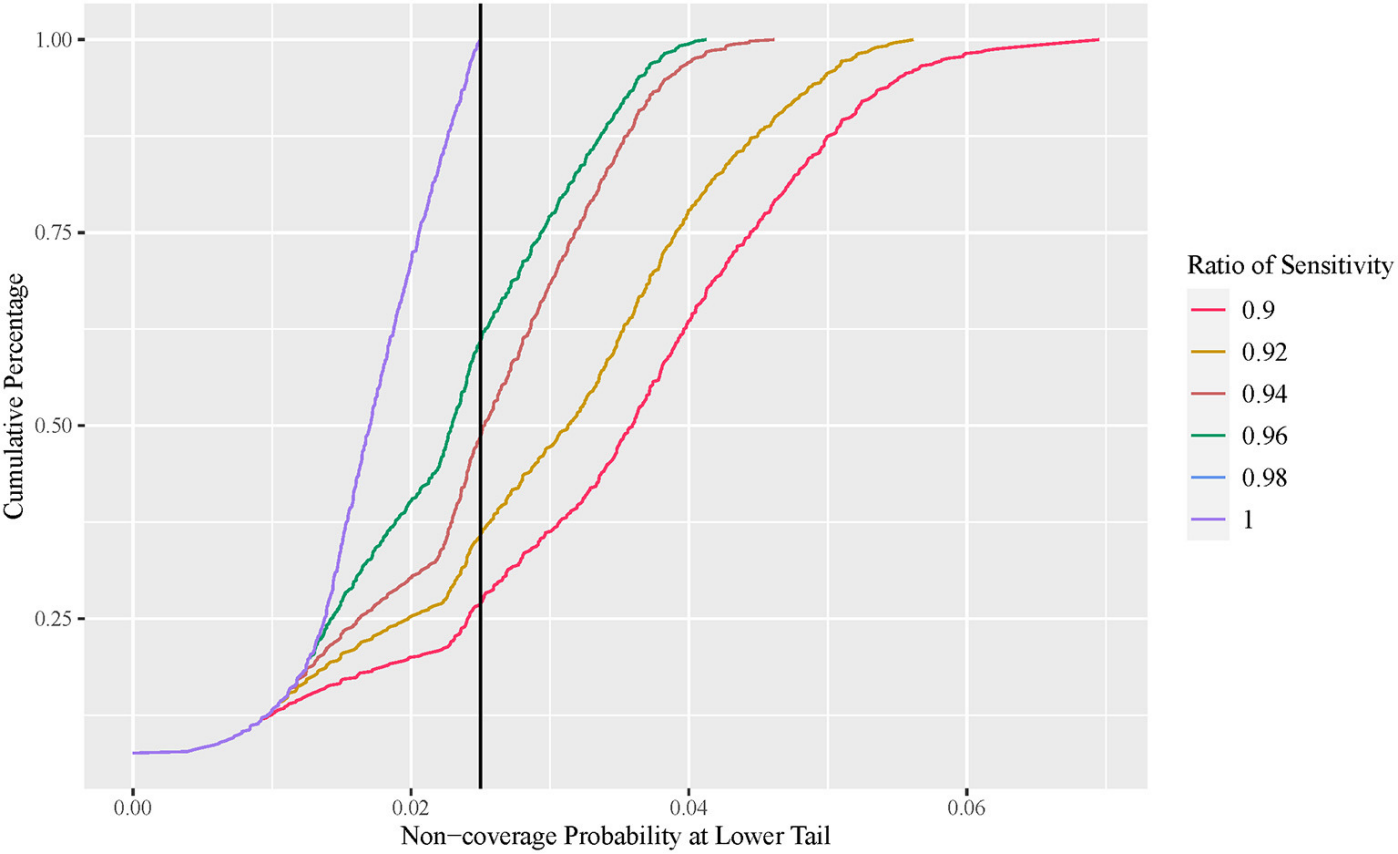
Cumulative percentage plot of one-sided non-coverage probability at lower tail with variable ratio of sensitivity (exact conditional method).

**Figure 5:**
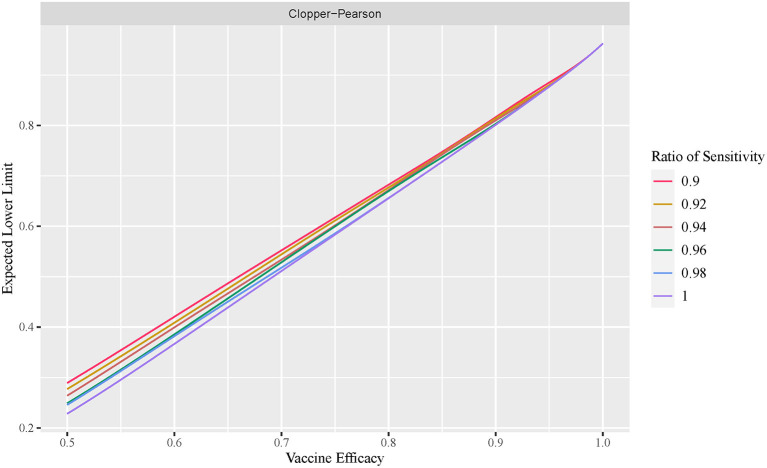
Expected lower limit of observed VE interval with variable ratio of sensitivity (exact conditional method).

The exact conditional method is often considered too conservative, with an unnecessarily wider interval width. In this section, we demonstrate that this is not always the case. Figure 4 plots the cumulative percentage of the non-coverage probability at the lower tail of the observed VE interval using the exact conditional method with a variable ratio of sensitivity. Figure 5 shows the expected lower limit of the observed VE interval with a variable ratio of sensitivity. As shown in Figure 4, when the ratio of sensitivity is below one, the non-coverage probability may exceed the nominal level 2.5%, and the lower the ratio of sensitivity, the higher the probability that the non-coverage probability exceeds the nominal level 2.5%. Moreover, Figure 5 shows that the lower the ratio of sensitivity, the higher the expected lower limit of the observed VE interval especially when the actual VE is close to 0.5. This indicates that the reported CI constructed using the number of observed cases tends to be overly optimistic when the ratio of sensitivity in the vaccine group to that in the placebo group is below one.

### Application to empirical data

In this section, we illustrate our results using the reported efficacy data for COVID-19 vaccines. Three studies were selected (see Example COVID-19 Vaccine Efficacy Trials), and their primary efficacy endpoint were analyzed using the five methods presented in this paper, with the results presented in Table 2.

**Table 2:** COVID-19 vaccine efficacy against symptomatic Covid-19 in three case studies.

**Category**	**Statistics**	**Placebo/control group**	**Vaccine group**
Example 1 (BNT162b2 vaccine)	N	17511	17411
	Number of cases	162	8
	Incidence rate per 1000 person-years	72.91	3.61
	Estimated VE%		95.0
	95% CI		
	Approximate Poisson method		89.9, 97.6
	Bayesian Jeffreys prior		90.5, 97.7
	Bayesian Pfizer prior		90.3, 97.6^†^
	Mid-p method		90.4, 97.7
	Exact Conditional method		90.0, 97.9
Example 2 (ChAdOx1 vaccine)	N	2025	2063
	Number of cases	33	12
	Incidence rate per 1000 person-years	156.98	56.24
	Estimated VE%		64.2
	95% CI		
	Approximate Poisson method		30.7, 81.5^†^
	Bayesian Jeffreys prior		32.3, 81.5
	Bayesian Pfizer prior		32.5, 81.8
	Mid-p method		31.8, 82.2
	Exact Conditional method		28.9, 83.2
Example 3 (HBO2 vaccine)	N	12737	12726
	Number of cases	95	21
	Incidence rate per 1000 person-years	44.70	9.80
	Estimated VE%		78.1
	95% CI		
	Approximate Poisson method		64.8, 86.3^†^
	Bayesian Jeffreys prior		65.4, 86.6
	Bayesian Pfizer prior		65.3, 86.6
	Mid-p method		65.3, 86.6
	Exact Conditional method		64.5, 87.0

N, number of participants in each group.

† The reported 95% CI for vaccine efficacy by the developer.

As indicated in Table 2, the VE interval constructed by the five methods are very similar when total number of infected cases is large (example 1). Among the five methods, the exact conditional method is always wider than the other methods, followed by the approximate Poisson method. The mid-p interval is slightly wider than the Bayesian intervals when the total number of cases is small (example 2).

Assume that the sensitivity of the diagnosis test is 90% in the vaccine group and 100% in the Placebo group, then the estimated VE% using actual number of cases in examples 1–3 are 94.5, 60.2, and 75.6, respectively. They are lower than the estimated VE% presented in Table 2, which indicates that the reported VE% and corresponding CI will be overly optimistic when the sensitivity of the diagnosis test in the vaccine group is lower than that in the Placebo group. The exact conditional method will be affected less than other methods due to wider interval width.

## Discussions

In this article, we present a few approaches to construct a CI for VE in a fixed number of events design. The approximate Poisson method is constructed using the Poisson distribution, while other approaches, including exact conditional interval, Bayesian intervals with Jeffreys prior or Pfizer prior, and mid-p interval, share a common feature in that they are all obtained by converting a CI for a single Binomial proportion.

### Exact conditional method

This method was converted from the Clopper-Pearson interval for a single proportion. This is the only method that guarantees two-sided coverage probability and non-coverage probability at the lower tail under all scenarios. The Clopper-Pearson interval is often considered to be too conservative for Binomial proportions (22–25), and Ewell (14) showed that the exact conditional interval for VE is overly conservative with a wide interval width (14). In our simulations, the exact conditional method produced an interval that was consistently wider than that of the other methods, especially when the total number of cases was small. When the total number of cases reaches 60, the interval width of all five methods are very similar.

### Mid-p method

This method was converted from the mid-p interval of a single proportion. Although the minimum coverage is below the nominal level, it exhibits slight oscillation among the methods except for the exact conditional method. The minimum coverage is closer to the nominal level, compared to approximate Poisson method and Bayesian methods. The mid-p interval is slightly wider than the Bayesian intervals and narrower than the approximate Poisson and exact conditional intervals.

### Approximate poisson method

The approximate Poisson method performs well when the VE is not close to one. When the VE is close to one, this method may lead to very low coverage, but non-coverage mainly comes from the upper tail. As a result, the lower limit of this method is conservative relative to the mid-p and Bayesian methods. This method provides a slightly narrower interval than the Clopper-Pearson method but wider than that of the other methods. Joshi, Geroldinger, and Jiricka et al. (26) showed that a Poisson interval does not exist when the number of infected cases is zero in the vaccine group (26).

### Bayesian method

The coverage probability of Bayesian methods with either the Pfizer prior or Jeffreys prior behaves more erratically than the other methods, and may lead to inflation of the type I error rate under some parameter settings because of low coverage.

In conclusion, we showed that the coverage probability for VE intervals is not fixed at the nominal significance level for all methods, due to the discreteness of the count data.

For the exact conditional method, it equals the nominal level or more while for the rest methods it may below the nominal level, even for a large number of total infected cases. In addition, our investigation shows that the exact conditional interval is too wide when the total number of infected cases is small, and hence, may not provide an informative CI. The narrower interval obtained using the Bayesian, mid-p, and approximate Poisson methods is at the cost of not preserving the nominal significance level. As a result, we suggest a mid-p method should be used when the total number of cases is below 60 to obtain a narrower interval width with a slight loss of coverage. When the total number of cases reaches 60, the exact conditional method has a similar interval width to that of the other methods without a loss of coverage.

Furthermore, our investigation of the effect of under-sensitivity of diagnosis testing shows that coverage probability decreases when the sensitivity in the vaccine group is lower than that in the placebo group. In such cases, the exact conditional method is preferred to guarantee coverage.

## Data availability statement

The raw data supporting the conclusions of this article will be made available by the authors, without undue reservation.

## Author contributions

QW conducted the study and wrote the main manuscript text under the instruction and oversight of PY. PW did a comprehensive review on the manuscript. All authors contributed to the article and approved the submitted version.

## Funding

The study was supported by the National Natural Science Foundation of China (No. 82173628) and the National Key R&D Program of China (No. 2018YFE0206900).

## Conflict of interest

The authors declare that the research was conducted in the absence of any commercial or financial relationships that could be construed as a potential conflict of interest.

## Publisher's note

All claims expressed in this article are solely those of the authors and do not necessarily represent those of their affiliated organizations, or those of the publisher, the editors and the reviewers. Any product that may be evaluated in this article, or claim that may be made by its manufacturer, is not guaranteed or endorsed by the publisher.
